# On the performances of Intensity Modulated Protons, RapidArc and Helical Tomotherapy for selected paediatric cases

**DOI:** 10.1186/1748-717X-4-2

**Published:** 2009-01-14

**Authors:** Antonella Fogliata, Slav Yartsev, Giorgia Nicolini, Alessandro Clivio, Eugenio Vanetti, Rolf Wyttenbach, Glenn Bauman, Luca Cozzi

**Affiliations:** 1 Oncology Institute of Southern Switzerland, Medical Physics Unit, Bellinzona, Switzerland; 2 London Regional Cancer Program, London Health Sciences Centre, London, Ontario, Canada; 3 Ospedale Regionale Bellinzona e Valli, Radiology Dept, Bellinzona, Switzerland

## Abstract

**Background:**

To evaluate the performance of three different advanced treatment techniques on a group of complex paediatric cancer cases.

**Methods:**

CT images and volumes of interest of five patients were used to design plans for Helical Tomotherapy (HT), RapidArc (RA) and Intensity Modulated Proton therapy (IMP). The tumour types were: extraosseous, intrathoracic Ewing Sarcoma; mediastinal Rhabdomyosarcoma; metastastis of base of skull with bone, para-nasal and left eye infiltration from Nephroblastoma of right kidney; metastatic Rhabdomyosarcoma of the anus; Wilm's tumour of the left kidney with multiple liver metastases. Cases were selected for their complexity regardless the treatment intent and stage. Prescribed doses ranged from 18 to 53.2 Gy, with four cases planned using a Simultaneous Integrated Boost strategy. Results were analysed in terms of dose distributions and dose volume histograms.

**Results:**

For all patients, IMP plans lead to superior sparing of organs at risk and normal healthy tissue, where in particular the integral dose is halved with respect to photon techniques. In terms of conformity and of spillage of high doses outside targets (external index (EI)), all three techniques were comparable; CI_90% _ranged from 1.0 to 2.3 and EI from 0 to 5%. Concerning target homogeneity, IMP showed a variance (D_5%_–D_95%_) measured on the inner target volume (highest dose prescription) ranging from 5.9 to 13.3%, RA from 5.3 to 11.8%, and HT from 4.0 to 12.2%. The range of minimum significant dose to the same target was: (72.2%, 89.9%) for IMP, (86.7%, 94.1%) for RA, and (79.4%, 94.8%) for HT. Similarly, for maximum significant doses: (103.8%, 109.4%) for IMP, (103.2%, 107.4%) for RA, and (102.4%, 117.2%) for HT. Treatment times (beam-on time) ranged from 123 to 129 s for RA and from 146 to 387 s for HT.

**Conclusion:**

Five complex pediatric cases were selected as representative examples to compare three advanced radiation delivery techniques. While differences were noted in the metrics examined, all three techniques provided satisfactory conformal avoidance and conformation.

## Background

Approximately fifty percent of paediatric cancer patients receive radiotherapy as part of their oncologic management [1]. In this population, balancing the potential for early and late toxicity against tumour control is particularly important. IMRT has been shown in several instances to improve conformal avoidance when compared to 3D conformal techniques and its role was investigated in a previous study on the same group of patients [2] and by many other authors [3-9]. Despite its potential, advanced photon treatments (mostly with IMRT) are still not widely used in the paediatric field as there is a substantial lack of knowledge on the late side effects [5]. The availability of more sophisticated techniques like intensity-modulated protons, helical tomotherapy and the newly introduced RapidArc, triggered interest in performing a new investigation to compare relevant dosimetric metrics when applied to paediatric cases.

Several pilot studies have studied the use of protons in paediatric radiation oncology [10-14] for various disease sites. In all cases a significant potential in terms of sparing of organs at risk, reduction of healthy tissue involvement and reduction of risk for secondary cancer induction was demonstrated. In comparing helical tomotherapy (HT) with other advanced photon delivery for cranial-spinal and extra-cranial irradiation, HT showed a superior degree of conformality [15-17]. Tempering these benefits, is the secondary neutron production by some proton techniques (passive scattering) and increased low dose radiated volumes for intensity modulated photon techniques that could contribute to an increase in second malignancies. Hall [18,19] suggested that children are more sensitive than adults by a factor of 10; in addition, there is an increased genetic susceptibility of paediatric tissues to radiation-induced cancer. Conversely, a recent publication from Schneider et al [20], estimating the relative cumulative risk in child and adult for IMRT and proton treatment with respect to conformal therapy, concludes that in the child, the risk remains practically the same for the two photon techniques or is reduced when proton therapy is used. This fact strengthen the interest in investigating new photon modalities in children cancer care.

In paediatric oncology, the variety of indications is large and, at the limit, every individual patient presents peculiarities preventing easy generalisations. As done in the previous investigation on IMRT [2], rather than selecting one single pathology and a consistent cohort of patients, we selected a small group of highly complex cases, presenting specific planning challenges regardless from the treatment intent and the actual stage of the diseases. The present study aims to address the problem of new technical solutions in paediatric radiation oncology: assuming that research activity in treatment planning, and not only at clinical level, should be promoted, it is important to analyse if the available tools could be adequate and effective also for those patients. Clinical potentials and outcomes should be addressed in clinical trials, and are not subject of comparative planning studies.

In the present paper a comparison among three highly sophisticated techniques has been carried out. No data have been reported here comparing IMRT, provided already in the previous publication [2] on the same group of patients, where different treatment planning systems where used; in that report, a conventional regime was used, but results would not substantially change on dosimetric comparison. In addition, comparison of also normal 3D-CRT (and IMRT) is not in the scope of this work because complex paediatric cases are not ideally planned with conventional approaches, while a clear preference is given to protons; RapidArc and Helical Tomotherapy could constitute and interesting intermediate level of standard, and aim of the present investigation is to understand their role with respect to the ideal solution of protons.

## Methods and patients

Five paediatric patients, affected by different types of cancer in different, challenging anatomic configurations were selected. The choice aimed to identify a group of difficult and challenging indications in terms of tumour location, anatomical boundary conditions, dose coverage, tolerance requirements. These cases might be also technical paradigm for other clinical indications with similar challenges.

A detailed summary of the indications, volume sizes, dose prescriptions and planning objectives is outlined in table 1. For all cases, except patient 5, the treatment was structured on two volumes to be concurrently irradiated by means of Simultaneous Integrated Boost approach: PTV1 being in general the elective and PTV2 the boost volumes. For patient 1 the boost volume was the surgical scar, not included in the elective volume and receiving a lower dose, while in patient 4 the boost volume excluded the inguinal nodes. The objectives concerning OARs refer mainly to the report of the National Cancer Institute [21,22]. Dose was normalised to the mean dose of the PTV volume receiving the higher dose prescription. The three following objectives were specified: *i) *target coverage (min. dose 90%, max. dose 107%), *ii) *OAR sparing to at least the limits stated in table 1, *iii) *sparing of Healthy Tissue (defined as the CT dataset patient volume minus the volume of the largest target).

**Table 1:** Main characteristics of patients and treatment plan.

	**Patient 1**	**Patient 2**	**Patient 3**	**Patient 4**	**Patient 5**
**Patient**	Male, 12 y.o.	Female, 8 y.o.	Female, 5 y.o.	Female, 13 y.o.	Female, 8 y.o.

**Diagnosis**	Ewing Sarcoma extraosseous, intrathoracic	Rhabdomyosarcoma mediastinum, stage III	Metastasis of base of skull with bone, para-nasal and lef eye infiltration from Nefroblastoma of right kidney	Rhabdomyosarcoma anus. Metastasis lymphnodes intrapelvic, inguinal and osseous	Wilm's tumour of the left kidney. (Multiple lung metastasis). Multiple liver metastasis

**Status**	After chemotherapy + surgery + chemotherapy	After chemotherapy	After chemotherapy + right nefrectomy	After chemotherapy	After chemotherapy + left nefrectomy + chemo-radiotherapy for lung metastasis

**Radiotherapy dose** **Prescription**	PTV = 28 × 1.9 = 53.2 Gy PTV scar = 28 × 1.6 = 44.8 Gy	PTVII = 25 × 1.98 = 49.5 Gy PTVI = 25 × 1.80 = 45.0 Gy	PTVII = 17 × 2.5 = 42.5 Gy PTVI = 17 × 1.8 = 30.6 Gy	PTVII = 25 × 1.98 = 49.5 Gy PTVI = 25 × 1.80 = 45 Gy	PTV = 15 × 1.2 18 Gy

**Target volumes**	PTV = 564 cm^3^ PTV scar = 14 cm^3^	PTVI = 109 cm^3^ PTVII = 72 cm^3^	PTVI = 1436 cm^3^ PTVII = 104 cm^3^	PTVI = 618 cm^3^ PTVII = 193 cm^3^	PTV = 1234 cm^3^

**Organs at risk dose objectives**	Lung^1 ^< 15 Gy Heart^1 ^< 30 Gy Vertebra^1 ^< 20 Gy Spinal cord^2 ^< 45 Gy	Lung^1 ^< 15 Gy Heart^1 ^< 30 Gy Vertebra^1 ^< 20 Gy Spinal cord^2 ^< 45 Gy	Right eye^1 ^< 40 Gy Left eye (blind)^1 ^< 50 Gy Lens^1 ^< 10 Gy Spinal cord^2 ^< 45 Gy	Rectum^1 ^< 40 Gy Bladder^1 ^< 30 Gy Uterus^1 ^< 20 Gy Femural heads^1 ^< 20 Gy	Kidney^1 ^< 10 Gy

**Techniques**	RA: 2 copl arcs, HDMLC HT: Fld s. 2.5 cm, pitch 0.43 IMP: 3 fields	RA: 2 copl arcs, HDMLC HT: Fld s. 2.5 cm, pitch 0.43 IMP: 2 fields	RA: 2 copl arcs, MLC120 HT: Fld s. 2.5 cm, pitch 0.43 IMP: 2 fields	RA: 2 non copl arcs, MLC120 HT: Fld s. 2.5 cm, pitch 0.43 IMP: 6 fields	RA: 2 non copl arcs, MLC120 HT: Fld s. 2.5 cm, pitch 0.43 IMP: 2 fields

**Delivery time** **MU**	RA: 129 s, MU: 479 HT: 387 s MU: NA IMP: NA MU: NA	RA: 123 s MU: 370 HT: 146 s MU: NA IMP: NA MU: NA	RA: 129 s MU: 538 HT: 341 s MU: NA IMP: NA MU: NA	RA: 127 s MU: 527 HT: 334 s MU: NA IMP: NA MU: NA	RA: 129 s MU: 483 HT: 255 s MU: NA IMP: NA MU: NA

1: mean dose; 2: maximum dose

The cases were selected in order to obtain a minimal set of complicated planning situations with specific challenges as described in [2] and summarized as follows:

For patient 1, the target was adjacent to the spinal cord, partially inside the lung with a long scar (about 5 cm) generating a secondary target volume, separated from the main one (smaller in volume) located along the thoracic wall and requiring simultaneous boost.

For patient 2, the location of the target in the mediastinum would be relevant in terms of large dose baths in the lung (and eventually breast) regions.

For patient 3, sparing of the right eye (the only functional) was the primary planning issue.

For patient 4, the target volume was divided into three separate regions (the anal volume and the two inguinal node regions) with organs at risk (uterus, bladder and rectum) generally positioned between the three targets.

For patient 5, the target volume was given by the entire liver and the main organ at risk was the right kidney with a low tolerance, located proximal/adjacent to the target. The sparing of this kidney had a very high priority since the patient underwent left nephrectomy.

### Planning techniques

#### RapidArc (RA)

RapidArc uses continuous variation of the instantaneous dose rate (DR), MLC leaf positions and gantry rotational speed to optimise the dose distribution. Details about RapidArc optimisation process have been published elsewhere by our group [23,24]. To minimise the contribution of tongue and groove effect during the arc rotation and to benefit from leaves trajectories non-coplanar with respect to patient's axis, the collimator rotation in RapidArc remains fixed to a value different from zero (from 20 to 45 degrees in the present study). This technicality permits to smear out the effect not having the interleaf space on the same axial position through the whole arc, that would transfer directly on the patient the tongue and groove effect.

All plans were optimised on the Varian Eclipse treatment planning system (TPS) (version 8.6.10) for a 6 MV photon beam from a Varian Clinac. The MLC used were either a Millennium with 120 leaves (spatial resolution of 5 mm at isocentre for the central 20 cm and of 10 mm in the outer 2 × 10 cm) or a High Definition (2.5 mm leaf width at isocentre in the central 8 cm region and 5 mm in the 2 × 7 cm outer region), depending on the target size (smaller volumes could benefit from High Definition MLC). Two arcs were applied, either coplanar or non coplanar. Details are reported in table 1. The Anisotropic Analytical Algorithm (AAA) photon dose calculation algorithm was used for all cases [25,26]. The dose calculation grid was set to 2.5 mm.

#### Helical Tomotherapy (HT)

During HT treatment, a 6 MV x-ray fan beam intensity-modulated by a binary multi-leaf collimator (MLC) is delivered from a rotating gantry while a patient is slowly moving through the gantry aperture resulting in a helical beam trajectory. A collimator aperture of 25 mm and a pitch of 0.43 were used for this study. The MLC is equipped with 64 leaves with a 0.625 cm width at isocentre. The gantry rotates at a constant speed while MLC leaves open 51 times per rotation and close entirely between different "projections". Plans were optimised using an inverse treatment planning process (based on least squares optimisation) determining MLC aperture times and the dose is calculated using a superposition/convolution approach. The software version used for this study was HiART TomoPlan 1.2 (Tomotherapy Inc., Madison, US). Details on the HT optimisation process can be found in [27,28]. Dose calculations were performed using the fine dose calculation grid (3 mm in cranio-caudal direction and over a 256 × 256 matrix in axial plane from the original CT scan, i.e. approximately 2 × 2 mm^2^)

#### Intensity Modulated Protons (IMP)

Intensity modulated proton plans were obtained for a generic proton beam through a spot scanning optimisation technique implemented in the Eclipse treatment planning system from Varian [29,30]. The simultaneous optimisation of the weight of each individual spot (from any number of fields) is performed inside a point cloud describing organs at risk and targets. Initial spot list is obtained at a pre-processing phase. In this phase, energy layers are determined which contain sets of spots located inside the target (plus eventual margins). Weight optimisation is performed starting from a dose deposition coefficient matrix calculated as the dose that would be deposited to each of the cloud points when irradiating each single spot of the initial list with a unit intensity. At the end of optimisation, a post-processing phase allows to prune unused energy layers as well as unused spots. The proton dose calculation algorithm used for the study was the version 8.2.22. The maximum energy available was 250 MeV with an energy spacing of 10 MeV between the layers. Applied nominal maximum energies ranged from 104 MeV (patients 2 and 4) to 152 MeV (patient 5). Spot spacing was set to 3 mm, circular lateral target margins were set to 5 mm, proximal margin to 5 mm and distal margin to 2 mm. Dose calculation grid was 2.5 mm. ln all cases coplanar beam arrangement was adopted using from 2 to 6 fields as specified in table 1.

#### Evaluation tools

All dose distributions were generated or imported (via DICOM) in the same treatment planning system (Eclipse), and from that the Dose-Volume Histogram (DVH) were exported to have all analysis based on DVH obtained with the same sampling algorithm.

Evaluation of plans was performed by means of standard DVH. For PTV, the values of D_99% _and D_1% _(dose received by the 99%, and 1% of the volume) were defined as metrics for minimum and maximum doses. To complement the appraisal of minimum and maximum dose, V_90%_, V_95%_V_107% _and V_110% _(the volume receiving at least 90% or 95% or at most 107% or 110% of the prescribed dose) were reported. The homogeneity of the treatment was expressed in terms of the standard deviation (SD) and of D_5%_–D_95% _difference. The conformality of the plans was measured with a Conformity Index, CI_90% _defined as the ratio between the patient volume receiving at least 90% of the prescribed dose and the volume of the PTV. To account for hot spots, the External volume Index (EI_D_) was defined as V_D_/V_PTV _where V_PTV _is the volume of the envelope of PTV's and V_D _is the volume of healthy tissue receiving more than the prescription dose. For OARs, the analysis included the mean dose, the maximum dose expressed as D_1% _and a set of appropriate V_X _and D_Y _values. For healthy tissue, the integral dose, "DoseInt", is defined as the integral of the absorbed dose extended over all voxels but excluding those within the target volume (DoseInt dimension is Gy*cm^3^). This was reported together with the observed mean dose and some representative V_x _values.

To visualise the difference between techniques, cumulative DVHs for PTV, OARs and healthy tissue, were reported with a dose binning of 0.05 Gy.

For RA and HT, delivery duration was reported in terms of beam-on time. Delivery time for IMP plans are not reported since the calculation model used in the study is not tailored to any specific treatment facility. Relevant technical parameters affecting delivery time (e.g. energy switch systems, magnetic deflectors, couch movements) cannot be simply generalised and could induce huge variations in actual beam on times.

## Results

**Figure 1:**
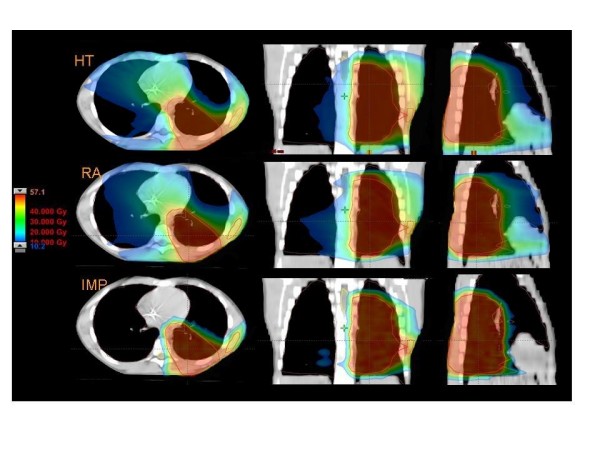
**Dose distributions in axial coronal and sagittal views for RA, HT and IMPT for Patient 1**.

**Figure 2:**
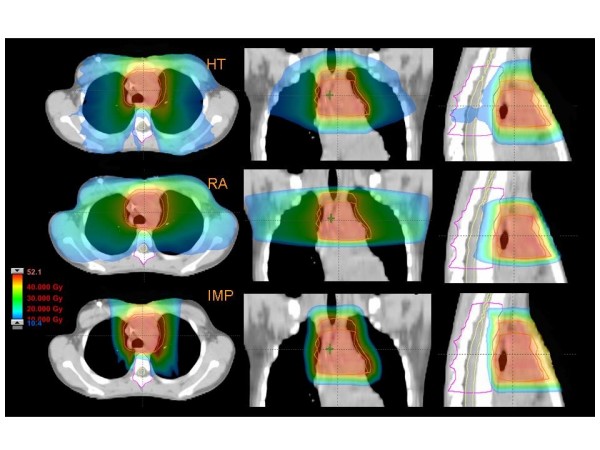
**Dose distributions in axial coronal and sagittal views for RA, HT and IMPT for Patient 2**.

**Figure 3:**
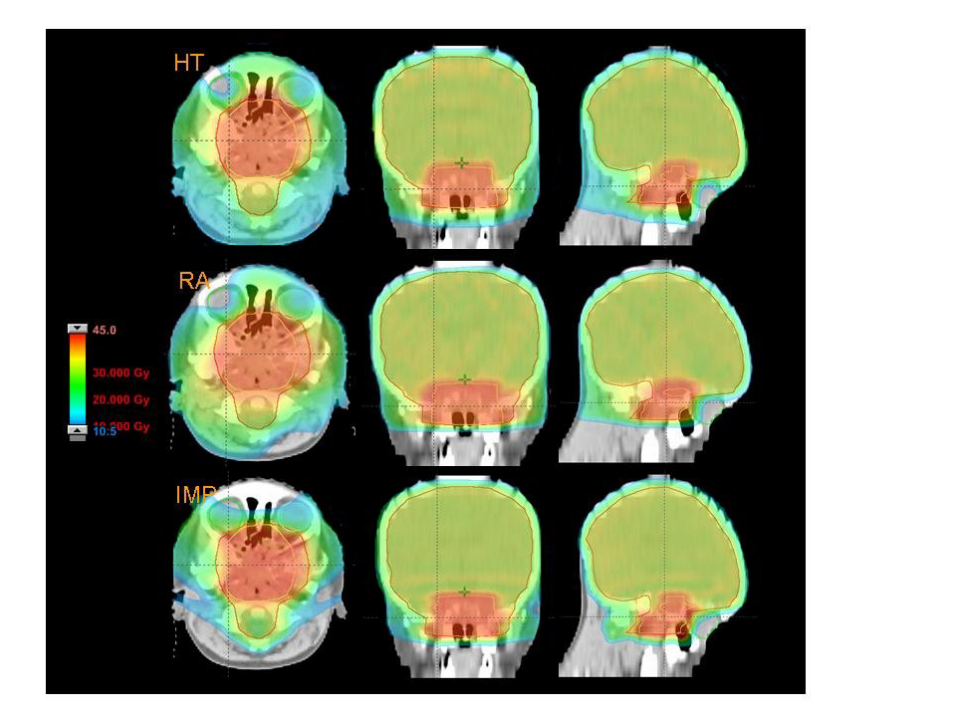
**Dose distributions in axial coronal and sagittal views for RA, HT and IMPT for Patient 3**.

**Figure 4:**
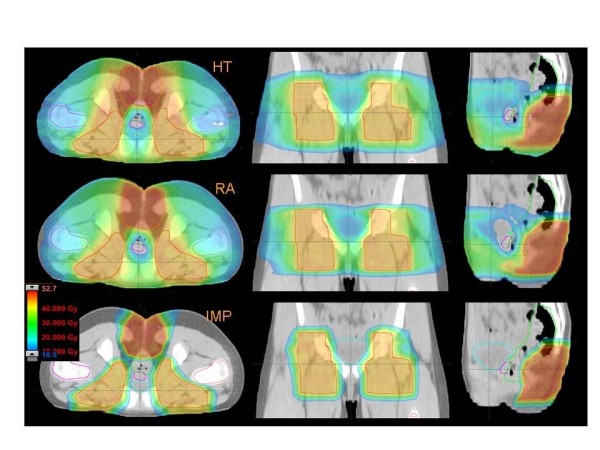
**Dose distributions in axial coronal and sagittal views for RA, HT and IMPT for Patient 4**.

**Figure 5:**
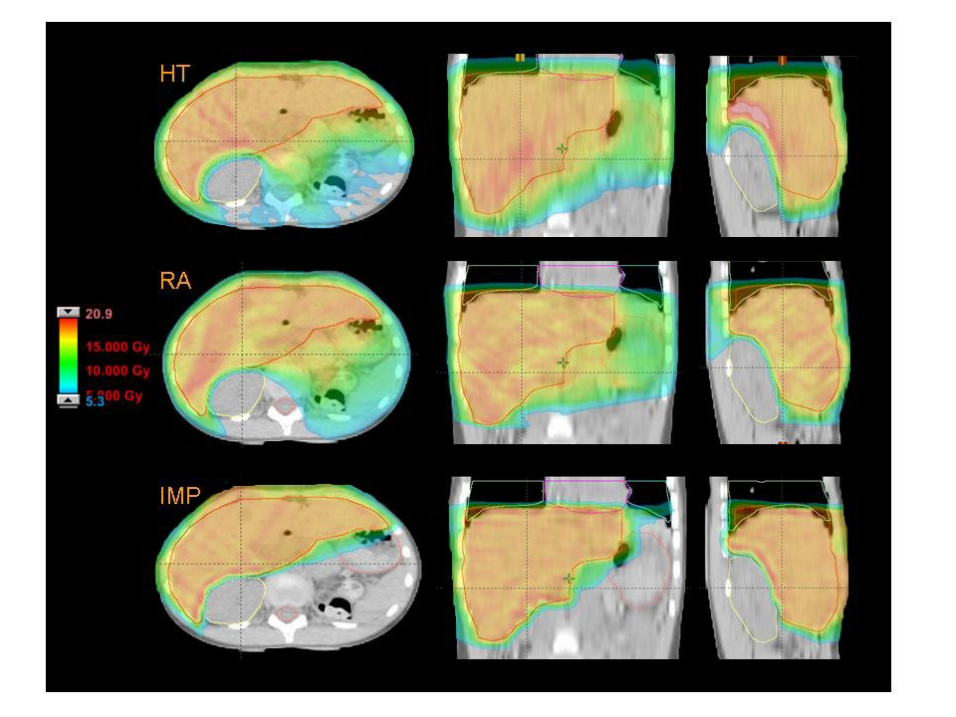
**Dose distributions in axial coronal and sagittal views for RA, HT and IMPT for Patient 5**.

Figures 1 to 5 present the dose distributions for our five patients for the three techniques. In each figure, axial, coronal, and sagittal views are shown to better appraise general characteristics of dose distributions (e.g target conformality and dose bath). The thresholds for the colour-wash representations are shown in the figures.

**Figure 6:**
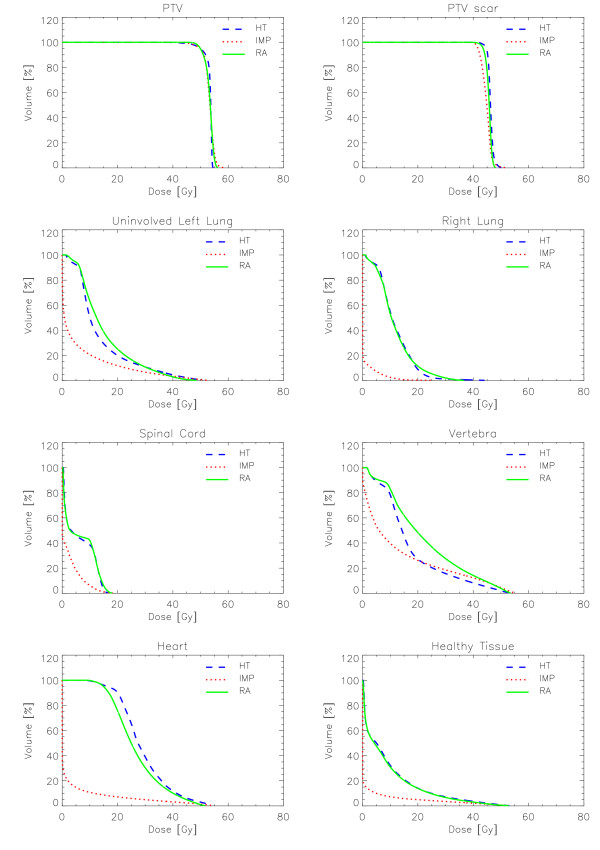
**Dose-Volume Histograms for targets and organs at risk for Patient 1**.

**Figure 7:**
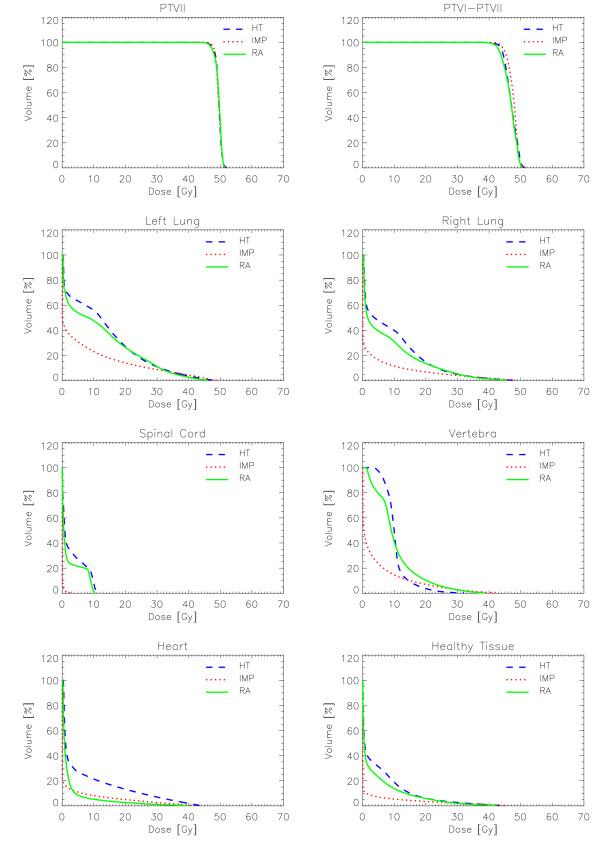
Dose-Volume Histograms for targets and organs at risk for Patient 2.

**Figure 8:**
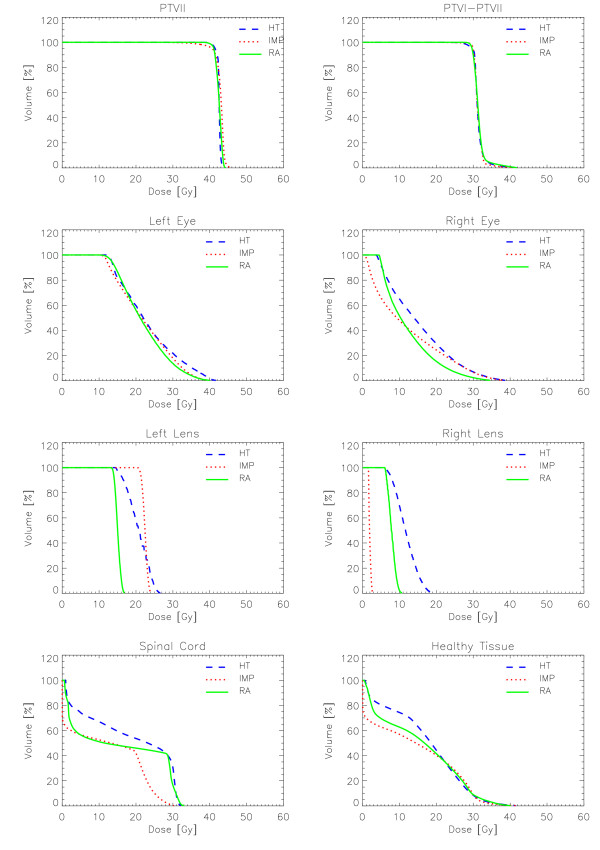
**Dose-Volume Histograms for targets and organs at risk for Patient 3**.

**Figure 9:**
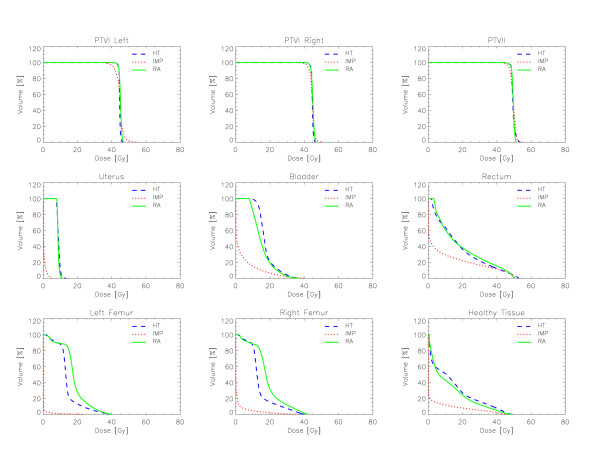
**Dose-Volume Histograms for targets and organs at risk for Patient 4**.

**Figure 10:**
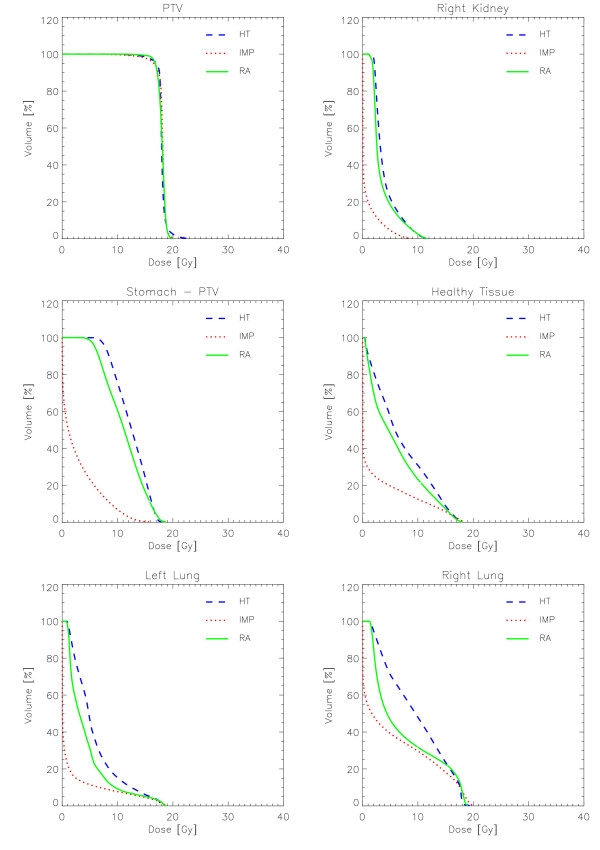
**Dose-Volume Histograms for target and organs at risk for Patient 5**.

Figures 6 to 10 show the DVHs of various target volumes, organs at risk and healthy tissue.

Tables 2 to 6 present a summary of the quantitative analysis performed on DVHs.

**Table 2:** Results from dose plan analysis for Patient 1

	**Obj**	**HT**	**IMP**	**RA**
***PTV***				
Mean [Gy]	53.2	53.2 (100%)	53.2 (100%)	53.2 (100%)
D_1% _[%]	-	102.3	107.1	104.9
D_99% _[%]	-	85.0	86.8	90.6
V_90% _[%]	100%	97.7	97.8	99.3
V_95% _[%]	100%	93.9	93.5	92.5
V_107% _[%]	0%	0.0	1.1	0.0
V_110% _[%]	0%	0.0	0.2	0.0
D_5_–D_95 _[%]	-	8.1	10.9	10.0
SD [%]	-	3.2	3.6	3.0
CI_90_	1.0	1.2	1.1	1.2

***PTV scar***				
Mean [Gy]	44.8	46.2 (103.1%)	44.7 (99.8%)	45.6 (101.8%)
D_1% _[%]	-	110.3	110.7	106.5
D_99% _[%]	-	96.0	90.6	94.0
V_90% _[%]	100%	99.9	99.4	99.9
V_95% _[%]	100%	99.5	88.3	98.4
V_107% _[%]	0%	5.3	3.0	0.3
V_110% _[%]	0%	1.2	1.2	0.0
D_5_–D_95 _[%]	-	7.1	12.3	7.8
SD [%]	-	2.5	4.0	2.5

***Vertebra***				
Mean [Gy]	< 20 Gy	17.6	13.6	22.2
D_1% _[Gy]	Minim	51.5	53.8	52.5

***Spinal Cord***				
D_1% _[Gy]	< 45 Gy	15.8	15.2	16.9

***Right Lung***				
Mean [Gy]	< 15 Gy	11.9	0.9	11.9
V_20 Gy _[%]	Minim	8.8	0.3	11.4

***Left uninvolved Lung***				
Mean [Gy]	< 15 Gy	14.1	6.4	15.5
V_20 Gy _[%]	Minim	20.0	11.8	25.1

***Heart***				
Mean [Gy]	< 30 Gy	29.1	3.8	26.7
V_40 Gy _[%]	Minim	11.9	3.2	10.1
D_1% _[Gy]	Minim	53.1	51.9	50.0

***Healthy tissue***				
Mean [Gy]	Minim	9.1	2.5	8.7
V_10 Gy _[cm^3^]	Minim	32.2	7.2	31.0
EI [%]	Minim	3.0	3.5	2.9
DosInt [10^5 ^*Gy*cm^3^]	Minim	0.84	0.23	0.81

**Table 3:** Results from dose plan analysis for Patient 2

	**Obj.**	**HT**	**IMP**	**RA**
***PTVII***				
Mean [Gy]	49.5	49.5 (100%)	49.5 (100%)	49.5 (100%)
D_1% _[%]	-	103.2	103.8	103.4
D_99% _[%]	-	93.3	94.7	92.7
V_90% _[%]	100%	99.9	100.0	99.8
V_95% _[%]	100%	97.5	98.7	96.2
V_107% _[%]	0%	0.0	0.0	0.0
V_110% _[%]	0%	0.0	0.0	0.0
D_5_–D_95 _[%]	-	6.5	5.9	7.3
SD [%]	-	1.8	1.8	2.2
CI_90_	1.0	1.1	1.1	1.0

***PTVI-PTVII***				
Mean [%]	45.0	47.0 (104.4%)	47.6 (105.8%)	46.7 (103.8%)
D_1% _[%]	-	112.9	113.1	111.3
D_99% _[%]	-	92.9	95.3	91.1
V_90% _[%]	100%	99.7	99.9	99.3
V_95% _[%]	100%	98.1	99.2	95.4
V_107% _[%]	0%	31.2	45.6	28.3
V_110% _[%]	0%	11.6	6.7	6.3
D_5_–D_95 _[%]	-	13.8	11.6	15.1
SD [%]	-	4.4	3.6	4.7

***Vertebra***				
Mean [Gy]	< 20 Gy	10.5	4.3	10.5
D_1% _[Gy]	< 45 Gy	25.8	37.2	34.8

***Spinal Cord***				
D_1% _[Gy]	< 45 Gy	11.0	1.4	10.1

***Right Lung***				
Mean [Gy]	< 15 Gy	9.1	3.6	7.7
V_5 Gy _[%]	Minim	47.7	16.5	43.7
V_20 Gy _[%]	Minim	14.8	6.9	13.6

***Left Lung***				
Mean [Gy]	< 15 Gy	13.2	7.0	12.0
V_5 Gy _[%]	Minim	63.7	30.9	54.0
V_20 Gy _[%]	Minim	27.0	14.0	26.4

***Heart***				
Mean [Gy]	< 30 Gy	6.5	2.4	2.3
V_20 Gy _[%]	Minim	13.2	5.0	2.7
D_1% _[Gy]	Minim	42.1	38.0	31.1

***Healthy tissue***				
Mean Gy]	Minim	5.0	1.7	4.0
V_10 Gy _[cm^3^]	Minim	18.9	5.4	8.9
EI [%]	Minim	0.0	0.1	0.0
DosInt [10^5 ^*Gy*cm^3^]	Minim	0.29	0.10	0.23

**Table 4:** Results from dose plan analysis for Patient 3

	**Obj.**	**HT**	**IMP**	**RA**
***PTVII***				
Mean [Gy]	42.5	42.5 (100%)	42.5 (100%)	42.5 (100%)
D_1% _[%]	-	102.4	106.1	103.5
D_99% _[%]	-	94.8	83.8	94.1
V_90% _[%]	100%	100.0	97.9	100.0
V_95% _[%]	100%	98.9	95.6	100.0
V_107% _[%]	0%	0.0	0.5	0.
V_110% _[%]	0%	0.	0.	0.
D_5_–D_95 _[%]	-	4.0	8.2	6.1
SD [%]	-	1.2	3.8	1.9
CI_90_	1.0	1.0	1.0	1.2

***PTVI-PTVII***				
Mean [Gy]	30.6	31.3 (102.3%)	31.3 (102.3%)	31.5 (102.9%)
D_1% _[%]	-	126.5	122.4	130.4
D_99% _[%]	-	89.9	95.8	94.8
V_90% _[%]	100%	98.9	99.6	100.
V_95% _[%]	100%	96.7	99.2	100.
V_107% _[%]	0%	9.7	6.6	8.3
V_110% _[%]	0%	5.4	2.9	5.2
D_5_–D_95 _[%]	-	13.4	9.5	14.0
SD [%]	-	5.2	4.6	5.6

***Spinal Cord***				
D_1% _[Gy]	< 45 Gy	31.8	29.4	32.3

***Right Eye***				
Mean [Gy]	< 15 Gy	15.3	12.2	12.3
V_20 Gy _[%]	Minim	29.2	24.3	15.3
D_1% _[Gy]	Minim	36.8	34.6	30.5

***Right Lens***				
Mean [Gy]	< 15 Gy	11.8	1.9	7.9
D_1% _[Gy]	Minim	18.4	2.7	10.2

***Healthy tissue***				
Mean Gy]	Minim	17.3	14.2	15.7
V_10 Gy _[cm^3]^	Minim	74.3	56.9	62.7
EI [%]	Minim	0.1	0.1	0.0
DosInt [10^5 ^*Gy*cm^3^]	Minim	0.25	0.20	0.23

**Table 5:** Results from dose plan analysis for Patient 4

	**Obj**	**HT**	**IMP**	**RA**
***PTVII***				
Mean [Gy]	49.5	49.5 (100%)	49.5 (100%)	49.5 (100%)
D_1% _[%]	-	106.9	106.5	103.2
D_99% _[%]	-	94.1	89.9	93.7
V_90% _[%]	100%	99.9	98.9	99.8
V_95% _[%]	100%	98.6	93.9	98.2
V_107% _[%]	0%	0.9	0.7	0.0
V_110% _[%]	0%	0	0	0
SD [Gy]	-	2.2	3.2	1.8
D_5_–D_95 _[Gy]	-	7.3	9.9	5.3
CI_90_	1.0	2.3	1.3	1.7

***PTVI (left and right)***				
Mean [Gy]	45	44.6 (99.1%)	44.6 (99.1%)	45.0 (100%)
D_1% _[%]	-	102.7	110.0	103.8
D_99% _[%]	-	94.0	85.6	94.4
V_90% _[%]	100%	99.8	96.8	99.9
V_95% _[%]	100%	98.5	87.5	99.0
V_107% _[%]	0%	0 0	6.2	0 0
V_110% _[%]	0%	0 0	1.4	0 0
D_5_–D_95 _[%]	-	4.0	13.6	5.6
SD [%]	-	1.6	4.2	1.8

***Uterus***				
Mean [Gy]	< 20 Gy	9.0	0.5	8.8
D_1% _[Gy]	Minim	11.4	4.4	10.7
V_10 Gy _[%]	Minim	12.4	0.0	4.4

***Rectum***				
Mean [Gy]	< 40 Gy	18.4	10.6	19.3
D_1% _[Gy]	Minim	52.2	51.0	49.9
V_40 Gy _[%]	Minim	12.4	11.8	15.4

***Bladder***				
Mean [Gy]	< 30 Gy	17.8	4.3	15.5
D_1% _[Gy]	Minim	34.3	35.0	33.0
V_20 Gy _[%]	Minim	21.0	6.5	19.2

***Right Femur***				
Mean [Gy]	< 20 Gy	14.4	1.5	18.8
D_1% _[Gy]	Minim	38.4	28.2	41.0

***Left Femur***				
Mean [Gy]	< 20 Gy	14,3	0.3	18.2
D_1% _[Gy]	Minim	37.2	10.8	37.7

***Healthy tissue***				
Mean Gy]	Minim	13.8	3.7	12.2
V_10 Gy _[cm^3]^	Minim	51.2	11.8	42.9
EI [%]	Minim	4.1	5.0	3.3
DosInt [10^5 ^*Gy*cm^3^]	Minim	1.1	0.3	1.0

**Table 6:** Results from dose plan analysis for Patient 5

	**Obj**	**HT**	**IMP**	**RA**
***PTV***				
Mean [Gy]	18.0	18.0 (100%)	18.0 (100%)	18.0 (100%)
D_1% _[%	-	117.2	109.4	107.8
D_99% _[%]	-	79.4	72.2	86.7
V_90% _[%]	100%	97.1	96.1	98.1
V_95% _[%]	100%	94.6	92.5	91.3
V_107% _[%]	0%	4.7	2.5	1.8
V_110% _[%]	0%	3.0	0.7	0.3
D_5_–D_95 _[%]	-	12.2	13.3	11.7
SD [%]	-	5.6	6.1	4.4
CI_90_	1.0	1.15	1.12	1.12

***Kidney***				
Mean [Gy]	< 10 Gy	4.0	0.8	3.5
D_1% _[Gy]	Minim	10.8	7.4	10.9
D_30% _[Gy]	Minim	4.1	0.2	3.5
V_5 Gy _[%]	Minim	22.0	4.6	18.4

***Stomach – PTV***				
Mean [Gy]	Minim	12.4	2.9	11.2
D_1% _[Gy]	Minim	17.4	13,8	17.8
D_30% _[Gy]	Minim	14.5	3.6	13.3
V_15 Gy _[%]	Minim	24.3	0.4	16.7

***Healthy tissue***				
Mean [Gy]	Minim	7.1	2.6	6.0
V_10 Gy _[%]	Minim	30.8	12.6	23.3
EI [%]	Minim	0.8	2.6	0.8
DosInt [10^5 ^*Gy*cm^3^]	Minim	2.77	1.03	2.36

Table 7 present the average over the five patients of the findings for the various target volumes and healthy tissue.

**Table 7:** Average results over the five patients from dose plan analysis on target volumes and healthy tissue.

	**Obj**	**HT**	**IMP**	**RA**
***PTVII***				
D_1% _[%]	-	106.4 ± 6.3	106.6 ± 2.0	104.6 ± 1.9
D_99% _[%]	-	89.3 ± 6.8	85.5 ± 8.5	91.6 ± 3.0
V_90% _[%]	100%	98.9 ± 1.4	98.1 ± 1.5	99.4 ± 0.8
V_95% _[%]	100%	96.7 ± 2.3	94.8 ± 2.4	95.6 ± 3.7
V_107% _[%]	0%	1.1 ± 2.0	1.0 ± 0.9	0.4 ± 0.8
V_110% _[%]	0%	0.6 ± 1.3	0.2 ± 0.3	0.1 ± 0.1
SD [Gy]	-	3.8 ± 2.6	5.0 ± 3.1	3.4 ± 1.5
D_5_–D_95 _[Gy]	-	6.6 ± 3.7	8.3 ± 4.0	7.4 ± 3.8
CI_90_	1.0	1.3 ± 0.5	1.1 ± 0.1	1.2 ± 0.3

***PTVI-PTVII***				
D_1% _[%]	-	113.1 ± 9.9	114.1 ± 5.7	113.0 ± 12.0
D_99% _[%]	-	93.2 ± 2.6	91.8 ± 4.8	93.6 ± 1.7
V_90% _[%]	100%	99.6 ± 0.5	98.9 ± 1.4	99.8 ± 0.3
V_95% _[%]	100%	98.2 ± 1.2	93.6 ± 6.5	98.2 ± 2.0
V_107% _[%]	0%	15.4 ± 13.9	15.4 ± 20.2	12.3 ± 14.4
V_110% _[%]	0%	6.1 ± 5.2	3.1 ± 2.6	3.8 ± 3.4
D_5_–D_95 _[%]	-	9.6 ± 4.8	11.8 ± 1.7	10.6 ± 4.6
SD [%]	-	3.4 ± 1.7	4.1 ± 0.4	3.7 ± 1.8

***Healthy tissue***				
Mean Gy]	Minim	10.5 ± 5.0	4.9 ± 5.2	9.3 ± 4.7
V_10 Gy _[cm^3]^	Minim	41.5 ± 21.7	18.8 ± 21.5	33.8 ± 20.3
EI [%]	Minim	1.6 ± 1.8	2.3 ± 2.2	1.4 ± 1.6
DosInt [10^5 ^*Gy*cm^3^]	Minim	1.1 ± 1.0	0.4 ± 0.4	0.9 ± 0.9

### Target coverage

From table 7, within the limits of averaging over patients with different characteristics, it can be seen that, for the PTV at highest dose prescription, RA presents slightly better D_1%_, D_99%_, V_90%_, V_107%_, V_110%_, SD; HT presents better V_95 _and D_5%_–D_95%_, and IMP presents lowest CI_90%_. The worst results for minimum dose and target coverage are typically observed for IMP due to the limits imposed in the optimisation phase to reduce at maximum high dose levels around the target and to reach high conformality. Concerning the outer target volumes PTVI-PTVII at lower dose prescription (corresponding to PTV scar in the first patient and PTVI left and right for patient 4) similar trends can be observed with RA showing best findings for D_1%_, D_99%_, V_90%_, V_107%_; HT for V_95%_, D_5%_–D_95% _and SD; IMP only for V_110%_. All techniques, if considered from a clinical perspective appear to be equivalent with a target coverage at V_90% _superior to 98% for the high dose volumes and to 92% for the low dose volumes, a heterogeneity (D_5%_–D_95%_) lower to 9% on the high dose volumes and a conformity index inferior to 1.3.

### Organs at risk

The different characteristics of patients prevent the possibility to draw average conclusions and therefore the analysis was done separate for the five cases.

#### Patient 1

All techniques respected the objectives on the spinal cord, heart and right lung. RA slightly failed to reach the planning objective for the vertebra and the uninvolved left lung. The latter is likely due to the lateral spread of doses in the low density medium physically not avoidable for photon beams of 6 MV and differently modelled by the convolution/superposition algorithms implemented in Eclipse and TomoPlan. It is unlikely that optimisation algorithm or hardware features of RA would be responsible of the effect that is not present in any other of the five cases (where mostly water equivalent tissues are present). Protons presented a significantly superior sparing of all OARs as clearly shown in the DVH figure. RA and HT are equivalent for the right lung and spinal cord; RA is moderately superior to HT for heart while HT better spares the left uninvolved lung.

#### Patient 2

All techniques respected planning objectives for this case. Compared to HT, RA showed a lower involvement of both lungs for doses below 20 Gy and a significantly lower involvement of heart at all dose levels (e.g 10.5% improvement for V_20 Gy_). HT is preferable for sparing the vertebra for doses higher than 10 Gy while RA is better below that level. No differences were observed for the spinal cord.

#### Patient 3

All techniques easily respected constraints on spinal cord and right lens. HT slightly violated the objective on the right eye. Figure 3 reports results also for the left eye (blind) that are of no clinical relevance but interesting to observe left-right "symmetry" of the different techniques.

#### Patient 4

The main challenge in this case was to minimize the exposure of organs at risk located inside the triangle formed by the three disconnected targets. Nevertheless all the techniques were able to largely improve the planning objectives. Concerning uterus both HT and RA attested mean dose below 10 Gy, more than a factor 2 below the constraint. Similarly for the Rectum (< 20 Gy for both RA and HT against an objective of 40 Gy), for the bladder (with a reduction of a factor ~2 for RA and ~1.7 for HT), and for the femurs. RA plans were generally better than HT for the bladder (below 20 Gy), equivalent to HT for the rectum and the uterus, and inferior to HT for the femurs. For this patient, IMP granted the most significant sparing of OARs compared to the photon techniques. The mean dose was about 10 times lower for the right and about 50 times lower for the left femurs; about 20 times lower for the uterus; about 4 times for the bladder and slightly less than a factor of 2 for the rectum.

#### Patient 5

For this patient, the primary planning objective was to protect the kidney and all techniques largely succeeded: HT and RA showed equivalent results (also visible from the DVH graphs) and IMP reduced of a factor about 5 the mean dose to this organ. RA resulted in a better sparing of the stomach and lungs although these organs were not explicitly considered in the optimisation phase and therefore no special effort was put in their sparing.

### Healthy tissue sparing

From table 7, IMP resulted systematically and significantly better than either RA or HT as expected reducing of a factor 2 the dose integral, the mean dose, and V_10 Gy_. Nevertheless, IMP showed a tendency to spill more dose outside the target volumes resulting in a higher External Index although inferior to 2.5%. It is important to notice the difference in V_10 Gy _between RA and HT (about 23% higher with HT), systematic effect due to the inherent wider dose penumbra in the cranial and caudal directions of HT. On a patient per patient basis and limiting to the two photon techniques (IMP being obviously the best), no differences from the DVH graphs can be observed between RA and HT for patient 1. For patient 2, RA granted slightly better sparing below 15 Gy if compared to HT. Similarly for patient 3, 4, and 5, RA additionally spared healthy tissue below 20 Gy.

### Delivery time

Beam-on times for RA and HT reported in table 1 range from 123 to 129 s for RA, average: 127.5 s (two arcs for each plan) and from 146 to 387 s for HT (average 292.6 seconds). The rather uniform time distribution of RA compared to the larger variation of HT is mostly due to the volumetric vs helicoidal delivery methods implying for HT a delivery time proportional to the target length (and inversely proportional to the pitch factor) while for RA there is an obvious independence from the target length.

## Discussion and conclusion

This study aimed to address the effectiveness of advanced radiation treatment techniques for selected challenging paediatric scenarios. The study compared Helical Tomotherapy, RapidArc and Intensity Modulated Protons. Each case was selected as paradigmatic of some planning challenge by virtue of the target to be treated and the surrounding anatomy/proximity of organs at risk and the study did not aimed to answer to a specific clinical issue but rather to investigate some dosimetric features of different techniques in a variety of conditions. All techniques sufficiently respected conformation and avoidance objectives and generated clinically acceptable plans. As expected, protons presented a significant improvement in OAR sparing at the price of a slightly compromised coverage of targets. This negative effect could be quite easily compensated by increasing, at planning level, the lateral, caudal and distal margins in the spot list creation and optimisation. In the present study the margins were set to rather tight values (between 2 and 5 mm), wider margins would impact on the sparing of OARs, on Conformity Index and External volume Index, but the space for advantageous trade-off is likely significant and the parameters adopted in the study do not affect the general conclusion about substantial superiority of protons. Nevertheless, since access to proton facilities is still relatively limited in the world and therefore, many paediatric patients will need photon based radiotherapy, it is of interest to explore advanced photon techniques. Restricting the discussion to HT and RA, it is possible to state that: *i) *both approaches are satisfactory and qualitatively comparable in terms of conformal avoidance; *ii) *as a consequence there is no evidence to prefer one solution over the other on a dosimetric base. That said, differences do exist between the two photon techniques. In first instance, HT dose distributions showed a systematically broadening of doses in the caudal and cranial directions compared to RA. This is due to the fact that HT plans were optimised using a field size (i.e. the width of the helicoidal slice) of 2.5 cm (at the time of the study, the 1.0 cm beam was not commissioned for the tomotherapy unit in London). In principle, a tighter conformation of doses along the cranial-caudal axis would have been possible using a field size of 1.0 cm but at the price of a significantly prolonged treatment time (this scales roughly inversely proportional to the field size). With the configuration used in this study the beam-on time for HT is 2.2 times longer than for RA. The clinical relevance of beam-on time is hard to quantify and is beyond the scope of this study but can impact on patient's comfort, stability of positioning and internal organs movement. In addition for very young patients requiring anaesthesia for treatment, shorter treatment times may be desirable to decrease the length of sedation (or in some cases perhaps avoid sedation).

The present study incorporated some assumptions or limits that shall be disclosed. A first issue might concern internal organs motion due to respiration, in principle important in patients 1, 2 and 5. All the techniques investigated here, do not currently allow for motion compensation through either gating or tumour tracking. This, up to now, has to accounted for with larger margins to targets. In any case, focussing on paediatric treatment, any solution that will arrive in the future will have to be carefully considered if longer treatment time will be needed to treat the patient (as it is for example with gating solutions). On the other side, studies proved that with photons, irradiation can be improved [31] if breathing control is applied, the same authors suggested that mid-ventilation phase could be an adequate surrogate of breath control since, statistically, it is the phase where targets can be "seen" by static beams for the longest time provided adequate margins are defined. In the present study, the CT dataset used can be considered as average mid-ventilation phase partially solving the issue. A recent investigation [32] proved also the principle feasibility of target tracking in combination with RA delivery. It is therefore possible to conclude that, in absence of advanced methods, mid-ventilation could be applied as a first degree approach waiting for the clinical implementation of tracking.

A second consideration is linked to the use of two arcs, some of them non co-planar, for the RA plans. Undoubtedly, the application of two arcs was necessary to achieve the high degree of conformal avoidance required by the planning objectives, also non co-planarity was introduced to improve results. Data for single arcs are not presented for simplicity. Since in the paediatric treatments, greatest care should be given to OARs sparing, the usage of multiple arcs offered a significant improvement and therefore was considered important. The option for the use of non-coplanar arcs is another difference between HT (which is coplanar in delivery) and RA and may offer advantages in some anatomic sub-sites [33].

Some limitations of the present study concern organs at risk not explicitly considered in the analysis. In particular: immature breasts for patient 2 or ovaries for patient 4 and were already addressed in the discussion of the previous publication [2]. Breast for patient 2: in this case the issue is not the involvement of the glands at high dose levels but rather the dose bath and the potential for secondary cancer induction. In the absence of reliable models to predict the risk of secondary cancers it would be mostly speculative to provide data pointing at this endpoint. Moreover there are no values in literature that can reliably be used as tolerance dose levels for breast irradiation (as an organ) in children. The breasts were anyway included in our analysis as part of the healthy tissue (instead of considering them as specific organs). For protons, a dorsal approach might have been eventually beneficial but, also with the geometry used in this study, the mean dose to the immature glands is anyway inferior to 5 Gy (being the glands mostly outside the field, figure 2) and therefore respecting tolerances often used for adults. Ovaries for patient 3: this is an even more delicate case since dose tolerance (in the range of 4–12 Gy) changes, decreasing with age. Ovaries were not included in the study because of their insufficient detection on the CT dataset. Given that the tolerance level is very low with respect to the prescribed dose (50.4 Gy), the impossibility to have a correct location of the organs, and their close proximity to the target, a proper sparing these organs would be unreliable and compromising too severely the target coverage. We recorded the dose to the 'ovarian region' resulting from the plans and it was around 20–30 Gy for photon techniques.

A final important point relates to the analysis of the healthy tissue involvement. Assuming the need to reduce maximally the amount of healthy tissue irradiated at any dose level, HT and RA was associated with significantly larger low dose volumes. This crucial difference is correlated directly to the risk of secondary cancer induction and, although no specific modelling was applied to the data, it is obvious, from the Hall [18,19], Cozzi [23], Ramsey [34] data on peripheral dose that, whenever possible, paediatric patients should be treated with IMP (spot scanning, not passive scattered). Beside the already addressed differences at the cranial-caudal edges of the target, HT and RA did not showed different patterns of healthy tissue irradiation and therefore can be associated with similar risks. Others have reported on comparisons of HT to other photon IMRT and demonstrated lower scattered dose [32]; as well, RA has been reported to be associated with lower scattered dose than other photon IMRT in [23]. This last point, together with the possibility of not increasing the risk for secondary cancers if technologies presenting a higher dose bath with respect to conformal treatment [20], proves the importance of considering such a modern techniques for children who need a radiation treatment.

In any case the clear minimal amount of low/medium dose level deliverable with protons is not reachable with photons, confirming the superiority of protons in terms of low dose spread. The integral dose to healthy tissue reported in table 7 as average over the five patients shows that this value for IMP is less than halved relatively to photon deliveries, and, in this respect, RA could result in slightly lower values than HT,

A remark on the choice of RA as a comparing technique used in the present work has to be clarified: this is one of the recently developed techniques based on linac, in the wider frame of the volumetric intensity modulated arc therapy. Other commercial solutions are becoming available nowadays. The results here shown, as they are, are clearly specific to RA, but similar general ideas could be eventually drawn also for the other intensity modulated arc solutions.

To conclude, the three techniques under investigation generated very similar plans for all the targets and no significant negative effect was observed in simultaneously optimising multiple dose levels. In particular the dose distributions of the differential targets (PTVII-PTVI) proved to be sharp with minimal tails (confirmed by relatively low findings on corresponding V_110%_). This fact is a guarantee of the high modulation and high dose gradient capabilities and precise conformation to dose prescription of three completely different optimisation approaches, volumetric (RA), helicoidal (HT) and voxelised (IMP).

All three methods are nicely adequate to generate very complex dose patterns and seem appropriate for further investigation in the context of clinical trials of advanced radiotherapy techniques for paediatric cancers.

## Competing interests

No special competing interest exists for any authors.

LC acts as head of research at Oncology Institute of Southern Switzerland and as Scientific Advisor at Varian Medical Systems AG, Zug, Switzerland.

## Authors' contributions

AF and LC designed the study. AF, GN, defined planning protocols and operative procedures.

RW defined volumes of interest. LC performed planning on Eclipse for RapidArc and Protons

SY, GB performed planning for Helical Tomotherapy. LC, AF, EV, AC and GN coordinated and carried out data collection, program development and statistical analysis LC wrote the manuscript.

All authors contributed read and approved the final manuscript.
